# Driving pressure during general anesthesia for minimally invasive abdominal surgery (GENERATOR)—study protocol of a randomized clinical trial

**DOI:** 10.1186/s13063-024-08479-x

**Published:** 2024-10-26

**Authors:** The GENERATOR–investigators, Galina Dorland, Tom D. Vermeulen, Markus W. Hollmann, Marcus J. Schultz, Liselotte Hol, Sunny G. L. H. Nijbroek, Jenni S. Breel–Tebbutt, Ary Serpa Neto, Guido Mazzinari, Lukas Gasteiger, Lorenzo Ball, Paolo Pelosi, Emre Almac, Maria P. Argente Navarro, Denise Battaglini, Marc G. Besselink, Patty E. M. M. Bokkerink, Janneke van den Broek, Marc P. Buise, Suzanne Broens, Zoë Davidson, Oscar Díaz Cambronero, Hannes Dejaco, Petra Y. Ensink-Tjaberings, Anna A. Florax, Marcelo Gama de Abreu, Marc B. Godfried, Matthew B. A. Harmon, Hendrik J. F. Helmerhorst, Ragnar Huhn, Robert Huhle, Wesley D. Jetten, Merijn de Jong, Joseph S. H. A. Koopman, Stephanie C. E. Koster, Dianne J. de Korte-de Boer, Geert-Jan A. J. M. Kuiper, Charlotte N. Laman Trip, Aurora M. Morariu, Stefan A. Nass, Gezina T. M. L. Oei, Alice C. Pap−Brugmans, Frederique Paulus, Jan-Willem Potters, Mandana Rad, Chiara Robba, Elise Y. Sarton, Sjoerd Servaas, Kirsten F. Smit, André Stamkot, Bram Thiel, Michel M. R. F. Struys, Thijs C. van de Wint, Jakob Wittenstein, Miriam Zeillemaker-Hoekstra, Tim van der Zwan, Sabrine N. T. Hemmes, David M. P. van Meenen, Nikolai Staier, Maximilian Mörtl

**Affiliations:** Department of Anesthesiology, Amsterdam University Medical Centers, Meibergdreef 9, Amsterdam, AZ 1105 The Netherlands

**Keywords:** Mechanical ventilation, Intraoperative ventilation, Driving pressure, Positive end − expiratory pressure, Recruitment maneuver, Minimally invasive abdominal surgery, Pulmonary complications, Postoperative complications, Postoperative pulmonary complications

## Abstract

**Background:**

Intraoperative driving pressure (ΔP) has an independent association with the development of postoperative pulmonary complications (PPCs) in patients receiving ventilation during general anesthesia for major surgery. Ventilation with high intraoperative positive end–expiratory pressure (PEEP) with recruitment maneuvers (RMs) that result in a low ΔP has the potential to prevent PPCs. This trial tests the hypothesis that compared to standard low PEEP without RMs, an individualized high PEEP strategy, titrated to the lowest ΔP, with RMs prevents PPCs in patients receiving intraoperative protective ventilation during anesthesia for minimally invasive abdominal surgery.

**Methods:**

“DrivinG prEssure duriNg gEneRal AnesThesia fOr minimally invasive abdominal suRgery (GENERATOR)” is an international, multicenter, two–group, patient and outcome–assessor blinded randomized clinical trial. In total, 1806 adult patients scheduled for minimally invasive abdominal surgery and with an increased risk of PPCs based on (i) the ARISCAT risk score for PPCs (≥ 26 points) and/or (ii) a combination of age > 40 years and scheduled surgery lasting > 2 h and planned to receive an intra–arterial catheter for blood pressure monitoring during the surgery will be included. Patients are assigned to either an intraoperative ventilation strategy with individualized high PEEP, titrated to the lowest ΔP, with RMs or one with a standard low PEEP of 5 cm H_2_O without RMs. The primary outcome is a collapsed composite endpoint of PPCs until postoperative day 5.

**Discussion:**

GENERATOR will be the first adequately powered randomized clinical trial to compare the effects of individualized high PEEP with RMs versus standard low PEEP without RMs on the occurrence of PPCs after minimally invasive abdominal surgery. The results of the GENERATOR trial will support anesthesiologists in their decisions regarding PEEP settings during minimally invasive abdominal surgery.

**Trial registration:**

GENERATOR is registered at ClinicalTrials.gov (study identifier: NCT06101511) on 26 October 2023.

**Supplementary Information:**

The online version contains supplementary material available at 10.1186/s13063-024-08479-x.

## Background

Postoperative complications of up to 40% have been reported in patients receiving ventilation during general anesthesia for major surgery [1–4]. Postoperative complications that involve the lungs, so–called postoperative pulmonary complications (PPCs), greatly increase length of hospital stay and even mortality [3]. Considering an estimated 55 million abdominal surgery procedures are performed globally each year, even a slight reduction in PPCs would have a significant impact on healthcare costs [4, 5].

Intraoperative lung–protective ventilation may prevent PPCs [6, 7]. Intraoperative driving pressure (ΔP) has an independent association with PPCs [8]. ΔP is dependent on positive end–expiratory pressure (PEEP) and recruitment maneuvers (RMs): when PEEP with RMs increases aerated lung tissue, ΔP will remain low or can even decrease. However, when PEEP with RMs causes overdistention of lung tissue, ΔP will increase. Accordingly, ΔP has been proposed as a digital biomarker for guiding intraoperative PEEP settings [8]. Currently, a trial named “Driving prESsure DurIng GeNeral AnesThesIa fOr open abdomiNal surgery” (DESIGNATION) tests whether individualized high PEEP with RMs targeting a low ΔP compared to standard low PEEP without RMs reduces the incidence of PPCs in patients planned for open abdominal surgery [9].

Over the past decade, minimally invasive abdominal surgery has become more popular than open abdominal surgery. The rapidly expanding group of patients undergoing minimally invasive abdominal surgery represents a challenging cohort, as both the Trendelenburg positioning and the pneumoperitoneum cause a cephalad shift of the diaphragm, potentially changing the effects of PEEP with RMs on the amount of aerated lung tissue [10]. Consequently, this could have an effect on the incidence of PPCs in this population. A recent worldwide prospective observational study showed that 65% of patients undergoing minimally invasive abdominal surgery are at increased risk for developing PPCs, and the association between intraoperative ΔP and PPCs was found to be much stronger in patients undergoing minimally invasive abdominal surgery compared to those undergoing open abdominal surgery [11, 12]. It remains uncertain whether the ventilatory approach tested in DESIGNATION could also prevent PPCs in patients undergoing minimally invasive abdominal surgery. ΔP can be reliably and reproducibly determined during pneumoperitoneum [12]. In addition, ΔP and intra–abdominal pressure (IAP) have a linear relationship. When IAP increases, ΔP will increase too [13]. Higher PEEP settings have the potential to (partially) prevent this increase in ΔP [14].

This randomized clinical trial, named “DrivinG prEssure duriNg gEneRal AnesThesia fOr minimally invasive abdominal SurgeRy” (GENERATOR), assesses whether individualized high PEEP, titrated to the lowest ΔP, with RMs reduces PPCs in patients undergoing minimally invasive abdominal surgery.

## Methods

### Objectives and design

**Fig. 1:**
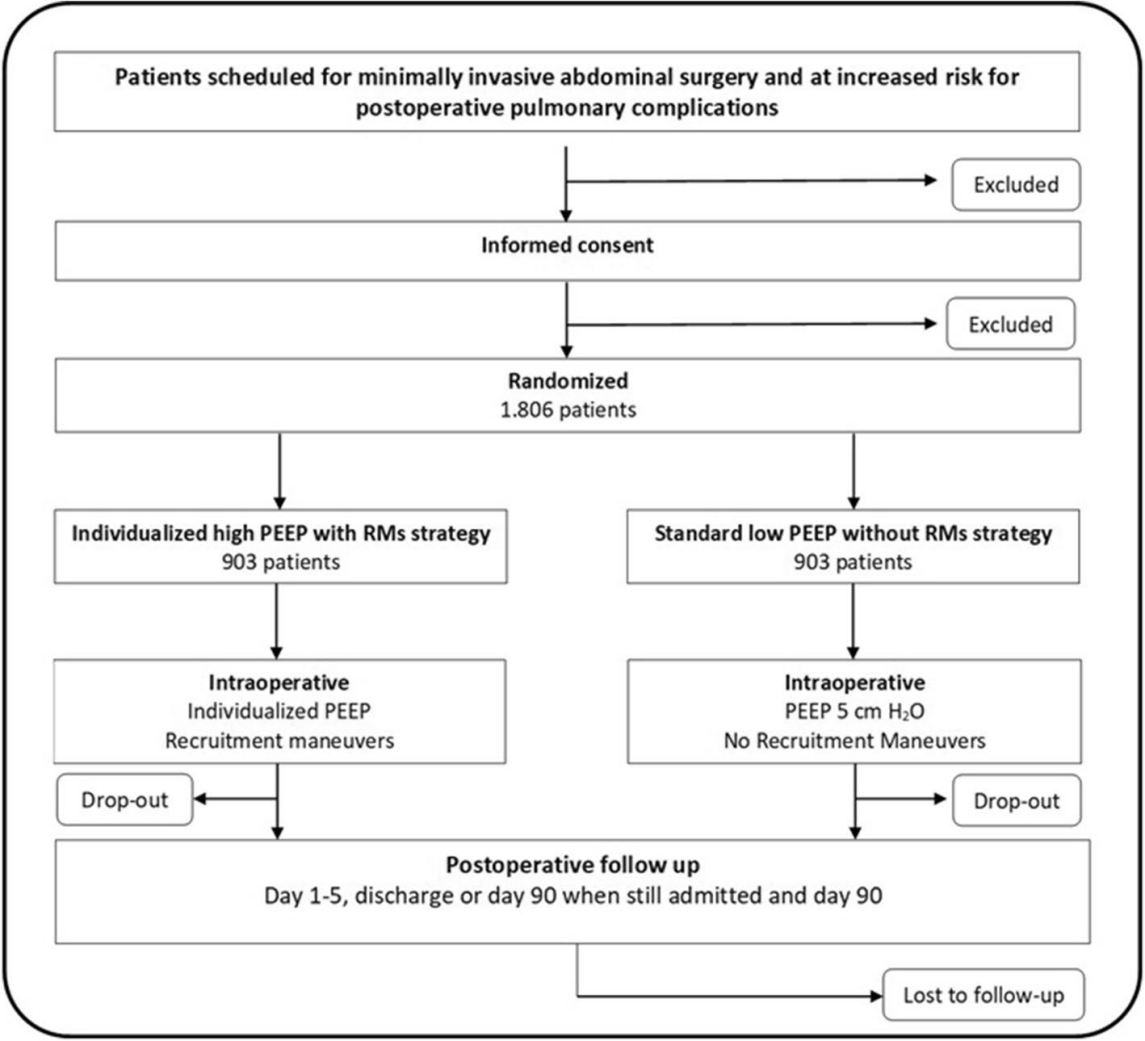
Consolidated Standards of Reporting Trials (CONSORT) diagram for the “DrivinG prEssure duriNg gEneRal AnesThesia fOr minimally invasive abdominal suRgery (GENERATOR)” trial

This investigator − initiated, international, multicenter, prospective, two–group, patient and outcome-assessor blinded randomized clinical trial (RCT) tests the hypothesis that in patients scheduled for minimally invasive abdominal surgery and at increased risk for PPCs, an individualized high PEEP strategy, titrated to the lowest ΔP, with RMs prevents PPCs when compared to a ventilation strategy that uses standard low PEEP without RMs. We further hypothesize that the individualized high PEEP strategy with RMs shortens duration of hospital stay and reduces the associated healthcare costs. A total of 1806 patients will be recruited in 20 academic and community hospitals in The Netherlands, Germany, Austria, Spain, and Italy (Table 4 in Appendix 1). Included patients will be randomly allocated in a 1:1 ratio to one of two intraoperative mechanical ventilation strategies (*see* Consolidated Standards of Reporting Trials (CONSORT) diagram in Fig. 1). The study design of GENERATOR is based on the ongoing DESIGNATION trial conducted by our group [9].

### Trial population

In order to be eligible to participate in this trial, a patient must meet the following criteria:

1. Age > 18 years; *AND*
2. Scheduled for minimally invasive abdominal surgery; *AND*
3. At increased (i.e., intermediate or high) risk of PPCs according to the “Assess Respiratory Risk in Surgical Patients in Catalonia” (ARISCAT) score (≥ 26 points, Table 1 [3]; *AND*/*OR* at increased risk of PPCs based on the combination of age > 40 years and scheduled surgery lasting > 2 h and planned to receive an intra–arterial catheter for blood pressure monitoring during the surgery [15]; *AND*
4. Signed written informed consent

**Table 1:** Assess Respiratory Risk in Surgical Patients in Catalonia scores

Risk for PPCs of variables selected for the logistic regression model			
	**Multivariate analysis**	*Β* coefficients	Risk score
	**OR (95% CI)** *N* = 1625		
Age, years			
≤ 50	1		
51–80	1.4 (0.6–3.3)	0.331	3
> 80	5.1 (1.9–13.3)	1.619	16
Preoperative SpO_2_, %			
≥ 96	1		
91–95	2.2 (1.2–4.2)	0.802	8
≤ 90	10.7 (4.1–28.1)	2.375	24
Respiratory infection in the last month	5.5 (2.6–11.5)	1.698	17
Preoperative anemia (≤ 10 g/dL)	3.0 (1.4–6.5)	1.105	11
Surgical incision			
Peripheral	1		
Upper abdominal	4.4 (2.3–8.5)	1.480	15
Intrathoracic	11.4 (4.9–26.0)	2.431	24
Duration of surgery, h			
≤ 2	1		
> 2 to 3	4.9 (2.4–10.1)	1.593	16
> 3	9.7 (4.7–19.9)	2.268	23
Emergency procedure	2.2 (1.04–4.5)	0.768	8

*SpO*_*2*_, oxyhemoglobin saturation by pulse oximetry breathing air in supine position

High or intermediate risk for postoperative pulmonary complications following abdominal surgery: ARISCAT risk score ≥ 26

Patients are excluded if they are scheduled for open abdominal surgery, combined abdominal and intra − thoracic surgery, or surgery in the prone or lateral position. Patients with a confirmed pregnancy or patients who consented for another interventional trial during anesthesia are not eligible. Other exclusion criteria are having received mechanical ventilation for longer than 30 min within the last 5 days prior to the current surgery, patients who are expected to require postoperative ventilation in the intensive care unit (ICU) or post–anesthesia care unit, expected hemodynamic instability or intractable shock and severe cardiac disease (New York Heart Association class (NYHA) III or IV, acute coronary syndrome (ACS) or persistent ventricular tachyarrhythmias). In addition, patients with a history of acute respiratory distress syndrome (ARDS), any major previous lung surgery (e.g., lung resection) and severe chronic obstructive pulmonary disease (COPD) with (noninvasive) ventilation or oxygen therapy at home or repeated systemic corticosteroid therapy for acute exacerbations of COPD are excluded as well.

### Standard ventilation management

In both groups, patients are ventilated in volume–controlled mode at the lowest possible inspired oxygen fraction (FiO_2_), with a minimum of 0.4, to maintain peripheral oxygen saturation (SpO_2_) above 90%. A pause time, between inspiration and exhalation, of 15% for each breath will be used. Inspiratory to expiratory ratio (I:E) is set at 1:2, and the respiratory rate is adjusted to target normocapnia (end–tidal carbon dioxide partial pressure between 35 and 45 mmHg [4.6 and 5.9 kPa]). Tidal volume (*V*_*T*_) is set at 8 ml/kg predicted body weight (PBW), calculated using a predefined formula: 50 + 0.91 × (centimeters of height − 152.4) for males and 45.5 + 0.91 × (centimeters of height − 152.4) for females. Ventilation management and the intervention described below are similar to the ongoing DESIGNATION trial [9].

### Intervention

**Fig. 2:**
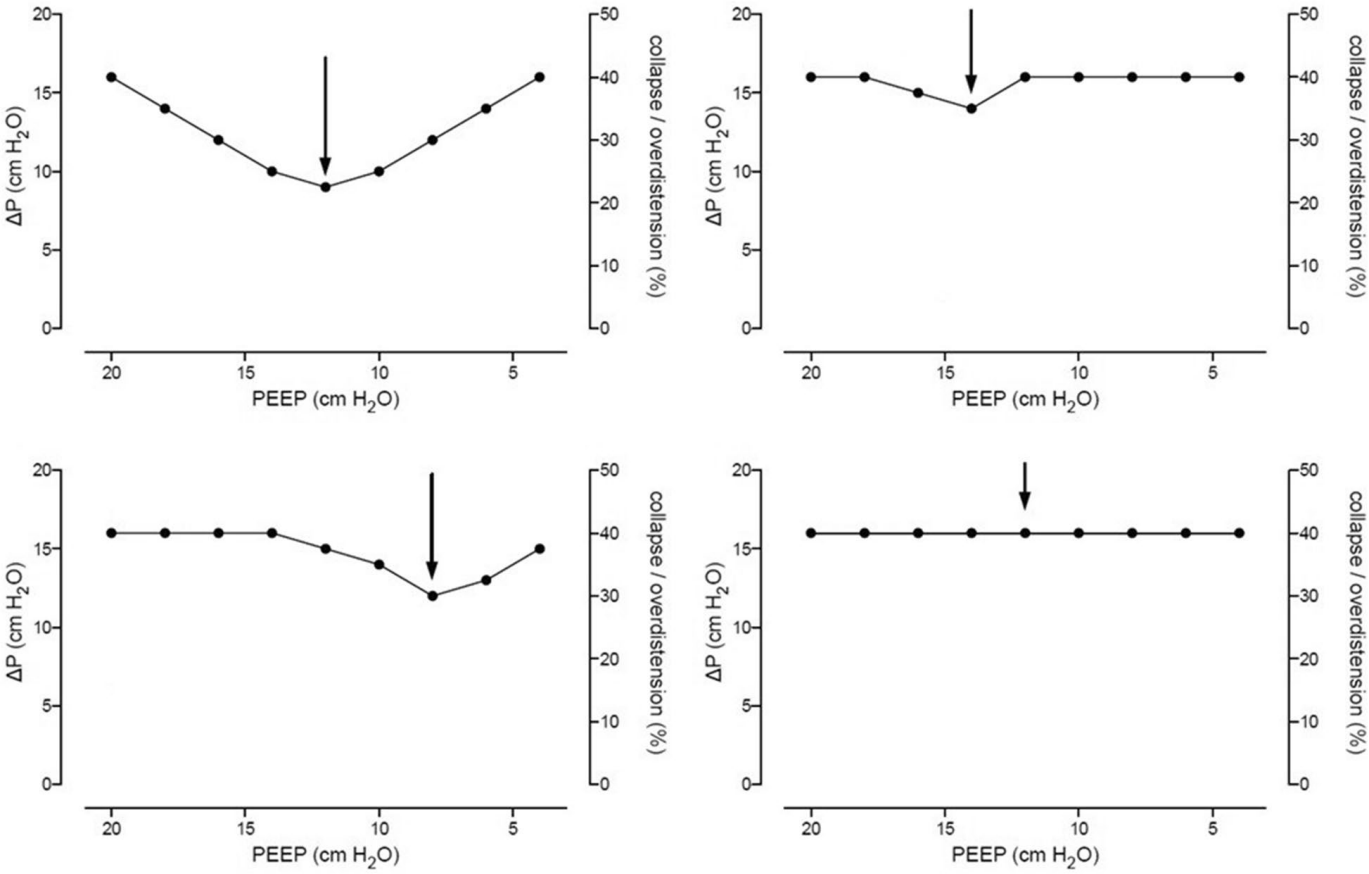
Examples of the “ΔP–PEEP” graph. The arrow represents the optimal PEEP

**Fig. 3:**
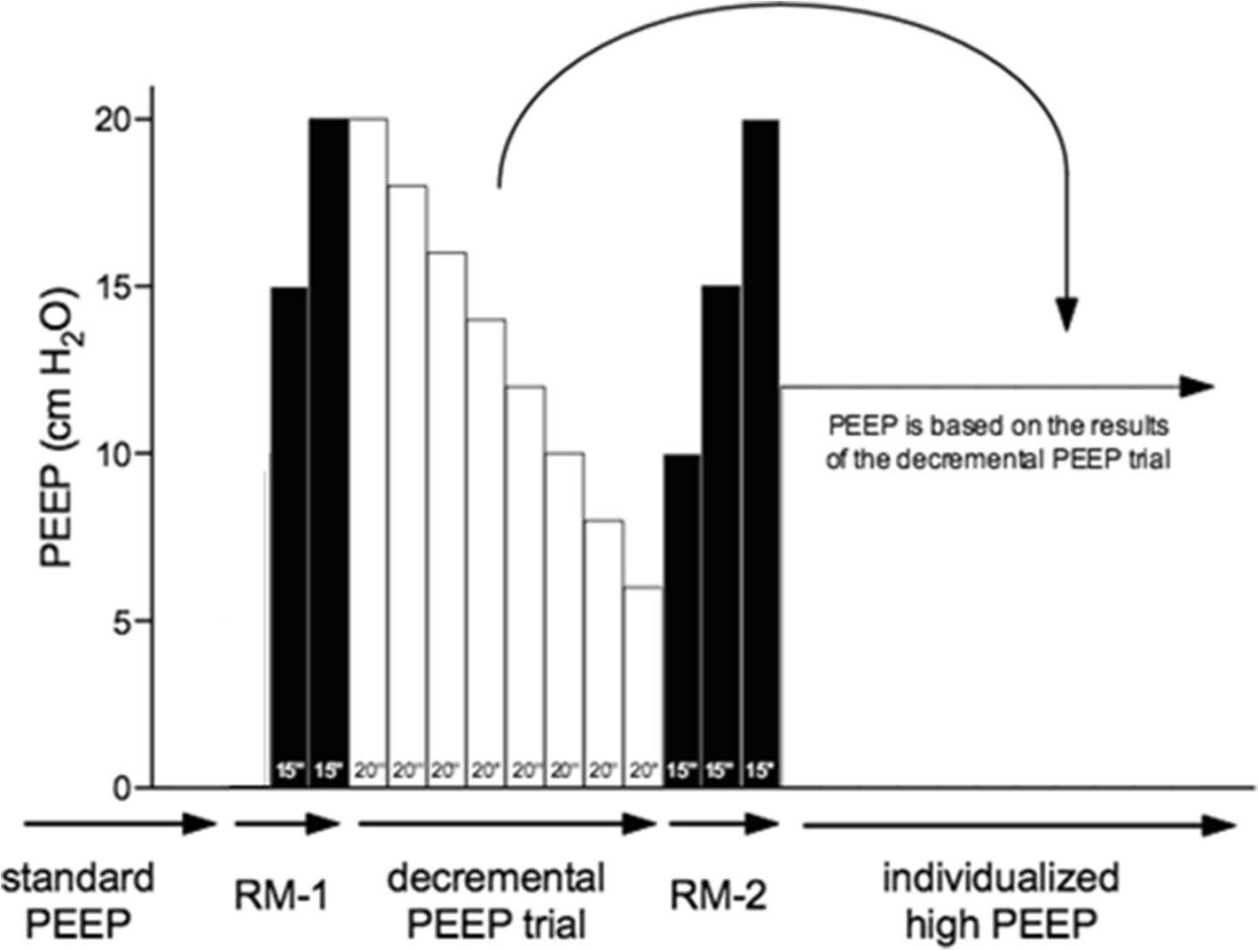
Overview of the intervention: the recruitment maneuvers and decremental PEEP trial. See text for a detailed description of the recruitment maneuvers and the decremental PEEP trial. The numbers projected in each bar represent the duration of each step in seconds

Patients randomized to the individualized high PEEP with RMs group will start with a PEEP of 10 cm H_2_O and will receive a RM followed by the decremental PEEP trial. RMs are conducted solely in a hemodynamically stable situation, as judged by the attending anesthesiologist. For this, the ventilator is kept in volume–controlled ventilation mode, with the respiratory rate set at 15 breaths per minute. In intervals of 15 s, PEEP is increased in steps of 5 cm H_2_O, starting at 10 cm H_2_O up to 20 cm H_2_O. The first RM will be conducted in the position in which the surgery will be performed, after abdominal insufflation and placement of all surgical instruments, i.e., in a steady state with sufficient working space for the surgeon. The surgeon is allowed to start surgery if no significant external pressure is applied on the abdomen and no new instruments are planned to be inserted. The RM will be repeated after any disconnection from the ventilator. The decremental PEEP trial is performed directly following the first RM, starting at a PEEP of 20 cm H_2_O with the respiratory rate set at 15 breaths per minute while the ventilator remains in volume–controlled ventilation mode. Every 20 s, PEEP is decreased in steps of 2 cm H_2_O until a minimum level of 6 cm H_2_O. Following each step, the resulting ΔP is calculated by subtracting PEEP from the plateau pressure (*P*_plat_). Next, a ΔP–PEEP graph is drawn by plotting ΔP against PEEP, as shown in Fig. 2. The highest level of PEEP with the lowest ΔP is determined from the ΔP–PEEP graph. If no clear nadir is present and the driving pressure is fluctuating within a 2-cm H_2_O difference at maximum, 12 cm H_2_O PEEP will be selected. The decremental PEEP trial is followed by a second RM, after which the individualized PEEP will be set, as determined by the decremental PEEP trial and maintained until the end of ventilation (Fig. 3).

### Conversion to laparotomy

In case of conversion to laparotomy, the PEEP will be set to 10 cm H_2_O during the conversion. After opening of the abdomen, the decremental PEEP trial will be repeated and the correct PEEP will be set until the end of surgery or until another radical change in patient position or intra–abdominal pressure.

### Control group

Patients randomized to the standard low PEEP without RMs group will receive 5 cm H_2_O PEEP for the complete duration of intraoperative ventilation. This specific PEEP level of 5 cm H_2_O has been selected as it represents the most commonly selected PEEP level in daily clinical practice [1, 16]. They will neither undergo the planned RMs nor a decremental PEEP trial.

### Rescue strategies

Desaturation (defined as SpO_2_ ≤ 90% or if preoperative SpO_2_ < 90% an absolute decrease in SpO_2_ > 5%) in patients receiving the individualized high PEEP with RMs strategy could reflect the presence of overdistention of aerated lung tissue, despite a low ΔP. If desaturations occur and there are no airway problems, severe hemodynamic impairment or ventilator malfunction, a rescue strategy is allowed. This strategy involves a stepwise reduction in PEEP and eventually increase of FiO_2_ (Table 2). In the standard low PEEP group without RMs, desaturation may indicate the presence of atelectasis. If desaturations occurs and there are no airway problems, severe hemodynamic impairment or ventilator malfunction, a rescue strategy is allowed by increasing FiO_2_ first, eventually followed by RM and PEEP increases (Table 3).

**Table 2:** Rescue for desaturation in the “individualized high PEEP with RMs” group

**Rescue for desaturation**		
**Step**	**PEEP**	**FiO**_**2**_
1	20	0.4
2	18	0.4
3	16	0.4
4	14	0.4
5	12	0.4
6	12	0.5
7	12	0.6
8	10	0.6
9	8	0.6
10	6	0.6
11	6	0.7
12	6	0.8

Down-titration of PEEP as rescue of desaturation. Starts at the level of PEEP set after the decremental PEEP trial

**Table 3:** Rescue for desaturation in the “standard low PEEP without RMs” group

**Rescue for desaturation**		
**Step**	**PEEP**	**FiO**_**2**_
1	5	0.4
2	5	0.5
3	5	0.6
4	5	0.7
5	5	0.8
6	6	0.8
7	RM	

Up-titration of PEEP and recruitment maneuvers (RM) as rescue of desaturation

### Preapproved protocol deviations

In both groups, the attending anesthesiologist is allowed to change ventilator settings at any time point upon the surgeons’ request or if concerns about patient safety arise. If one of the following complications occurs and does not respond to conventional therapy, PEEP can be changed, according to the judgement of the anesthesiologist in charge:

(i) After PEEP titration, a mean arterial pressure (MAP) < 65 mmHg, lasting > 1 min and not responding to fluids and/or vasoactive drugs [17];
(ii) New arrhythmias not responding to the treatment suggested by the Advanced Cardiac Life Support (ACLS) guidelines [18];
(iii) Need for a dosage of vasoactive drugs at the highest level tolerated, according to the decision of the anesthesiologist in charge;
(iv) Need of massive transfusion, more than five units of blood to maintain hematocrit > 21% and hemoglobin > 7 mg/dL; and
(v) Surgical complication resulting in life–threatening situation.

Any deviation from the protocol, excluding those mentioned above, are classified as protocol violations. Protocol violations are to be reported and will be discussed with the data safety monitoring board (DSMB).

### Standard procedures

Throughout the intraoperative period, routine elements of general anesthesia and surgery, including the intra-abdominal CO_2_ insufflation pressure and the use of neuromuscular blocking agents, are to be determined by the attending anesthesiologist and surgeon. Postoperative pain management, physiotherapeutic procedures, and fluid management will be administered according to each center’s specific expertise and routine clinical practice, ensuring minimal interference with the trial intervention. Quantitative neuromuscular monitoring, e.g., train-of-four (TOF) is required and residual curarization should be excluded prior to extubation (e.g., TOF > 0.9). It is strongly recommended to adhere to the Enhanced Recovery After Surgery (ERAS) guidelines.

### Minimization of bias

The local investigators perform randomization using the randomization tool in Castor Electronic Data Capture (EDC) [19]. Included patients will be randomly allocated in a 1:1 ratio to the individualized high PEEP with RMs group or the standard low PEEP group without RMs. The allocation sequence is generated by Castor EDC, uses permuted blocks with a maximum block size of 6, and is stratified per center and by body mass index (≤ 30 vs. > 30 kg/m^2^). Further minimization of bias will be achieved by involving at least two independent investigators per site. One investigator will be responsible for the preoperative and intraoperative trial period. A second investigator, who will remain blinded for treatment allocation, will score the primary and secondary postoperative outcome measures. Inherently to the type of intervention, the attending anesthesiologist is not blinded. Other caregivers, including the surgeon, research staff and ward nurses are blinded to treatment allocation.

### Trial endpoints

The primary endpoint of GENERATOR is a composite of PPCs until postoperative day 5 as used before in the DESIGNATION trial [9]. A patient meets the endpoint upon experiencing at least one PPC. The composite comprises the following PPCs:

(i) Mild respiratory failure, defined as the occurrence of one or multiple of the following conditions: peripheral oxygen saturation (SpO_2_) < 90% or partial pressure of oxygen in the arterial blood (PaO_2_) < 7.9 kPa (or < 50 mmHg) in room air, but responding to supplemental oxygen; a sudden increase in supplemental oxygen requirement to maintain adequate saturation (SpO_2_ > 90%) in patients receiving routine postoperative oxygen therapy; any level of supplemental oxygen after more than 2 days postoperatively;
(ii) Severe respiratory failure, defined as need for noninvasive or invasive mechanical ventilation, or a PaO_2_ < 60 mmHg (or < 7.9 kPa) or SpO_2_ < 90% despite supplemental oxygen in spontaneously breathing patients;
(iii) Bronchospasm, defined as newly detected expiratory wheezing treated with bronchodilators;
(iv) Suspected pulmonary infection, defined as receiving antibiotics and meeting at least one of the following criteria: new or changed sputum, new or changed lung opacities on chest radiograph when clinically indicated, tympanic temperature > 38.3 °C, white blood cell (WBC) count > 12,000/μL;
(v) Pulmonary infiltrate, defined as any unilateral or bilateral infiltrates on chest radiography;
(vi) Aspiration pneumonitis, defined as respiratory failure after inhalation of regurgitated gastric contents;
(vii) Atelectasis, defined as lung opacification with a shift of the mediastinum, hilum, or hemidiaphragm towards the affected area and compensatory overinflation in the adjacent non − atelectatic lung on chest radiography;
(viii) ARDS, according to the “Berlin definition for ARDS” [20];
(ix) Pleural effusion, defined as blunting of the costophrenic angle, loss of the sharp silhouette of the ipsilateral hemidiaphragm in upright position, evidence of displacement of adjacent anatomical structures or (in supine position) a hazy opacity in one hemithorax with preserved vascular shadows on chest radiography;
(x) Cardiopulmonary edema, defined as clinical signs of congestion, including dyspnea, edema, rales, and jugular venous distention, with the chest radiograph demonstrating increase in vascular markings and diffuse alveolar interstitial infiltrates; and
(xi) Pneumothorax, defined as air in the pleural space with no vascular bed surrounding the visceral pleura on chest radiography.

The secondary endpoints include:

(i) Intraoperative complications, that are not related to induction or change of the depth of anesthesia, consisting of:
  ◦ Any episode of desaturation, defined as SpO_2_ ≤ 90% or if preoperative SpO_2_ < 90% an absolute decrease in SpO_2_ > 5% and lasting > 1 min;
  ◦ Any episode of hypotension defined as MAP 65 < mmHg and lasting > 1 min [17];
  ◦ Any need for vasoactive agents, either as bolus or continuous administration, defined as more than needed to compensate for vasodilating effects of anesthesia as judged by the attending anesthesiologist;
  ◦ Any new arrhythmias needing intervention as suggested by the ACLS guidelines [18];
(ii) Intraoperative fluid strategy, including the total amount of fluids administered during anesthesia, including the amounts of colloids, crystalloids, and blood products;
(iii) Postoperative extrapulmonary complications (*see* Appendix 2);
(iv) All–cause mortality at day 5, day 30 and 90 after surgery and in − hospital mortality; and
(v) Cost–effectiveness parameters including:
  ◦ Presence and duration of postoperative supplemental oxygen;
  ◦ Use of antibiotics for pneumonia;
  ◦ Occurrence of imaging (chest x–ray; computed tomography scan);
  ◦ Length of stay in hospital; and
  ◦ Unplanned admission to an intensive care unit (ICU) (and if applicable, length of ICU admission).

### Trial visits and data collection

Patients are visited both preoperatively and intraoperatively. Daily follow − up is conducted during the initial five postoperative days or until discharge, whichever comes first. If patients remain hospitalized for more than 5 days after surgery, an additional visit will be conducted upon hospital discharge. The last day of follow − up is defined as day 90 after surgery. Consequently, patients who are still hospitalized will be contacted for the last time 90 days after their surgery (*see* Table 5 in Appendix 3).

All eligible patients will be informed about the trial and asked for written informed consent before surgery. Sufficient time will be provided to the patients to consider their participation. During the preoperative visit, baseline variables are collected including sex, age, height, weight, functional status (independent, partially independent or totally independent), physical status (according to the American Society of Anesthesiologists score), cardiac status (heart failure, according to the NYHA score, ACS, or persistent ventricular tachyarrhythmias) and smoking status. Data on pulmonary status, COPD (if inhalation therapy and/or systemic steroids are used) and respiratory infection within the last month before surgery will be collected as well. Other collected baseline variables include history of active cancer, history of diabetes mellitus including the use of insulin or oral antidiabetics, type of scheduled surgery, transfusion of blood products within 6 h prior to surgery and vital parameters (SpO_2_ and blood pressure). Blood tests (creatinine, hemoglobin, white blood cell count) and chest imaging (assessed on mono − and bilateral infiltrate, pleural effusion, atelectasis, pneumothorax, and cardiopulmonary edema) are collected solely when deemed necessary for clinical care for the patient.

After induction of anesthesia, variables are collected hourly during the intraoperative period. These variables include ventilator settings, vital parameters, intra-abdominal pressure, administered fluids and vasoactive drugs, blood transfusions, need for rescue strategy for hypoxemia, intraoperative complications possibly related to PEEP titrations, preapproved protocol deviations and violations, details of anesthesia (type, epidural analgesia, neuromuscular function monitoring), patient positioning during surgery, arterial blood gas results (only if deemed necessary for clinical care for the patient), amount of fluid loss, and duration of both surgery and anesthesia procedures. Patients allocated to the individualized high PEEP with RMs group will have their plateau pressure recorded at the end of each decremental PEEP trial step and a ΔP–PEEP graph will be drawn.

Postoperatively, clinical data and the occurrence of pulmonary and extrapulmonary postoperative complications are collected. Blood tests and chest imaging will only be performed when considered necessary for the patients’ clinical care. Life status (death or alive) will be recorded during the first five postoperative days and at day 90 after surgery. Total length of stay in hospital, unplanned admission to an intensive care unit (ICU), and length of stay in the ICU are captured as well. To assess the health − related quality of life, the validated and commonly used EQ − 5D − 5L questionnaire will be sent out by email at day 90 after surgery to Dutch patients.

### Trial dropouts and missing data

Because participation in the trial is voluntary, patients can leave the trial at any time for any reason if they wish to do so without any consequences. No or minimal losses to follow − up for the primary and secondary outcomes are anticipated. Complete–case analysis will be carried out for all the outcomes, that is, excluding patients with missing data in the outcome of interest. However, if any missing data is found for the primary outcome, a sensitivity analysis using multiple imputations and estimating − equation methods will be performed.

### Handling of data

All patient’s identifiable personal data will be separated from the research data and replaced by an assigned code. Data will be obtained from the electronic patient record and entered into the electronic case report form stored in Castor [19], a good clinical practice − compliant web–based data management system. The handling of personal data adheres the general data protection regulations and applicable national laws. After completing the data collection, full access to the database will be granted to selected investigators. Data are restricted available after analyses and publication of the main paper. All data will be stored in a secure place for 15 years after study end. The results of GENERATOR will be published in scientific journals and used for national and international guidelines. A summary of the results will be placed on ClinicalTrials.gov to inform participants.

### Sample size calculation

The required sample size is calculated based on an estimated effect size derived from individual patient data from previous clinical trials [6, 21, 22]. Based on a recently published randomized clinical trial [15], we conservatively estimate an incidence of PPCs of 30% in the standard low PEEP without RMs group. To have a power of 80% to detect a relative risk reduction in the incidence of PPCs of 20% (24% vs 30%), given an alpha of 0.05, 860 patients in each group are needed. Assuming a dropout rate of 5%, 903 patients per group are needed, resulting in a total sample size of 1806 patients.

### Statistical analysis

Before completion of recruitment, a complete statistical analysis plan will be made available online. All statistical analyses will be conducted according to the modified intention–to–treat (ITT) principle considering all patients in the treatment groups to which they were randomly assigned, excluding those lost to follow–up due to consent withdrawal or surgery cancelation. Baseline characteristics for both arms will be presented as counts and percentages, means and standard deviations (SD), or medians and interquartile ranges (IQR), depending on the normality of data distribution. Hypothesis tests will be two–sided with a statistical significance level of 5% (i.e., *p* < 0.05) for all outcomes. No adjustments will be made for the *p*–value for multiple comparisons. Statistical analysis will be performed using the free software program “R” (R Core Team, 2020, Vienna, Austria).

### Trial organization

The steering committee is composed of the principal investigator, the coordinating investigator, the local Amsterdam UMC investigators and five international experts of ventilation who contribute to the design and revisions of the trial protocol. The steering committee will be responsible for interpreting the data and drafts the final report that will be approved by all investigators. A DSMB, consisting of renowned, independent anesthesiologists (Arthur Bouwman, Idit Matot, John Laffey, Francesca Rubulotta), guards the ethics of conducting the trial in accordance with the Declaration of Helsinki and monitors safety and the overall conduct of the trial. The DSMB will meet after 25%, 50%, and 75% of patients are included or at least within 9 months after the first patient is enrolled. All unexpected non–trial related (S)AEs will be reported to the DSMB. Trial–related SAEs will be sent to the DSMB, as soon as possible but at latest within 7 days after being received by the coordinating center.

This trial is an investigator–initiated trial, funded by “The Netherlands Organization for health Research and Development” (ZonMw) and sponsored by the Amsterdam UMC, location Academic Medical Center (AMC). The sponsor has the authority to suspend the trial if there is sufficient ground that continuation of the trial may compromise the health or safety of the patients.

A qualified monitor will be assigned by the clinical monitoring center of the Amsterdam UMC to oversee the trial in accordance with the approved monitoring plan. This monitor will perform on-site as well as remote monitoring. Monitoring responsibilities include verifying inclusion rate, proper documentation and execution of informed consent, proper use of inclusion and exclusion criteria, and review of the source documents, as described in the monitoring plan. Centralized initiation meetings will be organized before sites can start including patients.

A complete checklist of recommended items to address in a clinical trial protocol and related documents according to the “Standard Protocol Items: Recommendations for Interventional Trials (SPIRIT) 2013” is provided (*see* Additional file 1).

## Discussion

The GENERATOR trial will be the first adequately powered multicenter, randomized clinical trial to investigate whether an individualized high PEEP strategy, titrated to the lowest ΔP, with RMs protects from PPCs in patients receiving protective ventilation during general anesthesia for minimally invasive abdominal surgery.

While mechanical ventilation is essential during general anesthesia for surgery, it is not without risks [23, 24]. Cyclic lung recruitment and overdistension are two mechanisms that contribute to the lung injurious effects of mechanical ventilation, potentially leading to PPCs [25]. Currently, there is general consensus that lung protective ventilation, involving low tidal volume and the use of PEEP, should be applied in patients receiving mechanical ventilation to minimize the risk of ventilator − associated lung injury and PPCs. The exact role of PEEP and RMs, however, remains unsure.

Previous large international randomized clinical trials failed to show a benefit in clinical outcomes of ventilation with high PEEP compared to low PEEP in abdominal surgery [21, 26]. A meta − analysis using individual patient data indicated that a rise in ΔP increases the risk of PPCs, as ΔP depends on the amounts of atelectatic and overdistended lung tissue, and suggested that PEEP should be titrated to ΔP [8].

Subsequently, a meta − analysis in patients receiving one lung ventilation during thoracic surgery reported that ΔP − guided PEEP was both feasible and effective in reducing PPCs [27]. However, there is still insufficient evidence to establish the effect of ΔP − guided PEEP on clinical outcomes in abdominal surgery. A randomized clinical trial from 2018 failed to demonstrate benefit from a personalized PEEP strategy on the development of PPCs in open abdominal surgery [28]. Although this trial did find a lower incidence of PPCs in the individualized PEEP group, the development of PPCs was one of the secondary endpoints, making the study underpowered to draw meaningful conclusions. Another randomized clinical trial demonstrated that ΔP − guided PEEP reduces PPCs in patients undergoing open abdominal surgery, although the sample size of 134 patients is too small to be conclusive [29]. As a result, the ongoing DESIGNATION trial, a sufficiently powered multicenter, randomized clinical trial, currently assesses an individualized high ΔP − guided PEEP with RMs strategy in patients undergoing open abdominal surgery [9].

Minimally invasive surgery techniques such as laparoscopic and robotic procedures have become increasingly popular. During minimally invasive abdominal surgery, the combination of Trendelenburg positioning and pneumoperitoneum causes a cephalad shift of the diaphragm, thereby affecting respiratory mechanics and increasing alveolar collapse, especially in lung parts close to the diaphragm [10]. Higher PEEP settings could have the potential to counterbalance these atelectasis − inducing effects and reduce PPCs. A recently published post hoc analysis found that the association between ΔP and PPCs is even stronger in patients undergoing laparoscopic rather than open abdominal surgery [12]. Therefore, a well powered multicenter, randomized clinical trial investigating a ΔP − guided PEEP strategy in patients undergoing minimally invasive abdominal surgery with relevant clinical endpoints is warranted.

In GENERATOR, a decremental PEEP trial will be conducted to determine the level of PEEP that results in the lowest ΔP possible. The decision to conduct a decremental PEEP with RMs trial derives from the ongoing DESIGNATION trial [9], in which an interim safety analysis indicated the feasibility of the intervention and demonstrated that the ΔP − guided PEEP with RMs resulted in a lower ΔP [30]. If no nadir in the ΔP is present, PEEP will be set at 12 cm H_2_O as previous studies have demonstrated that this level results in maximum lung opening during intraoperative ventilation, regardless of the FiO_2_ [21, 31–35]. For the standard low PEEP without RMs group, a PEEP level of 5 cm H_2_O has been chosen based on previous clinical trials on protective mechanical ventilation [9, 28, 36]. While there is no absolute consensus on the optimal PEEP level, a PEEP level of 5 cm H_2_O is the most commonly selected PEEP level in daily clinical practice [1, 16]. All patients in GENERATOR will receive mechanical ventilation with a low tidal volume (i.e., 8 mL/kg PBW). This approach has been shown to be safe in previous studies and has been used in prior clinical trials of intraoperative ventilation, enabling a robust comparison of the GENERATOR results with those from preceding trials [7, 9, 15, 21]. For both groups, rescue therapies are permitted and pre − approved protocol deviations are allowed to safeguard patients while maintaining a standardized approach that minimizes the interference with the respective interventions. Patients with supraglottic devices will not be included in the trial, as we anticipate potential leakage during RM and PEEP titration in these patients.

The main strengths of the current study are the large sample size, multicenter approach and pragmatic protocol design. GENERATOR will be conducted in both academic as non − academic hospitals in different countries in Europe, making the results generalizable. The main outcome measure, which is a composite of PPCs, is a clinically relevant endpoint that has been used in several clinical trials on intraoperative ventilation [21, 26, 28], as PPCs can be combined due to their shared underlying pathophysiological mechanisms. In addition, even so − called minor pulmonary complications are associated with an increased risk of mortality and length of hospital stay in surgical patients [4]. Therefore, the composite endpoint of PPCs has clinical significance and enhances the statistical power of the trial due to summation of incidences of single events. In GENERATOR, both the composite endpoint and the individual incidences of each specific PPC will be reported separately. Additionally, the trial will assess the impact of the intraoperative ventilation strategy on the occurrence of extrapulmonary complications as well as the length of stay in hospital and ICU. These secondary endpoints hold both clinical relevance and are essential to estimate the related health care costs.

An important limitation of the trial is that, due to the nature of the intervention, blinding of the attending anesthesiologist is not possible. Patients and postoperative assessors, however, will be fully blinded to the intraoperative period. Further minimization of bias will be achieved by involving at least two independent investigators per site, concealed allocation and avoiding loss to follow–up. Differences in clinical care practice such as intra–operative fluid management, administration of neuromuscular blocking agents and postoperative pain management could be potential confounding factors but are expected to be equally distributed over both groups due to the randomized design of the trial. These factors are not protocolized as GENERATOR aims to be a pragmatic trial. As per the protocol, perioperative care will be administered according to the specific expertise and routine clinical practices of each center. However, to minimize the influence of clinical care on the study intervention, suggestions on perioperative procedures have been made in the protocol. No specific recommendations are made on type of anesthesia to use, making the trial as accessible as possible for anesthesiologists. Since commonly known risk factors for PPCs regarding perioperative care will be reported and collected, it will be possible to assess their effects on both the primary and secondary endpoints.

In conclusion, GENERATOR is a multicenter, patient and outcome-assessor blinded, randomized clinical trial to test the hypothesis that compared to standard low PEEP without RMs, an individualized high PEEP, titrated the lowest ΔP possible, with RMs prevents PPCs in patients planned for minimally invasive abdominal surgery. The results of GENERATOR will support anesthesiologists in their decisions regarding intraoperative PEEP settings during protective ventilation for general anesthesia in minimally invasive abdominal surgery.

## Trial status

The current approved version of the protocol is version 2.0, issue date: 8 February 2024. Recruitment started on 11 December 2023. The estimated study completion date is December 2027.

## Supplementary Information

Additional file 1. SPIRIT 2013 Checklist: Recommended items to address in a clinical trial protocol and related documents.

## Data Availability

The full trial protocol and informed consent materials are available on any reasonable request to the corresponding author. The datasets analyzed during the present study are available on a reasonable request after approval of the steering committee. A full monitoring plan is obtainable on reasonable request.

## Appendix 1

**Table 4:** GENERATOR investigators

Site name	Collaborator(s)
Department of Anesthesiology, Amsterdam University Medical Centers, Amsterdam, the Netherlands	Hollmann, Markus W. Schultz, Marcus J. van Meenen, David M.P. Dorland, Galina Vermeulen, Tom D. Hol, Liselotte Nijbroek, Sunny G.L.H. Breel–Tebbutt, Jenni S.
Department of Anesthesiology, Albert Schweitzer Hospital, Dordrecht, the Netherlands	Jetten, Wesley D. Stamkot, G. André Ensink-Tjaberings, Petra Y.
Department of Anesthesiology, HAGA Hospital, The Hague, the Netherlands	Rad, Mandana van der Zwan, Tim de Jong, Merijn
Department of Anesthesiology, Onze Lieve Vrouwe Gasthuis, Amsterdam, the Netherlands	Godfried, Marc B. Thiel, Bram Davidson, Zoë
Department of Anesthesiology, Spaarne Gasthuis, Haarlem and Hoofddorp, the Netherlands	Servaas, Sjoerd
Department of Anesthesiology, University Medical Center Groningen, Groningen, the Netherlands	Struys, Michel M.R.F. Zeillemaker-Hoekstra, Miriam
Department of Anesthesiology, Maastricht, University Medical Center + , Maastricht, the Netherlands	Buise, Marc P. Kuiper, Geert-Jan A.J.M. Dianne, Korte – de Boer
Department of Anesthesiology, Dijklander Hospital, Hoorn, the Netherlands	Oei, Gezina T.M.L Smit, Kirsten F.
Department of Anesthesiology, Isala Zwolle, Zwolle, the Netherlands	Morariu, Aurora M. Pap − Brugmans, Alice C. Bokkerink, Patty E.M.M.
Department of Anesthesiology, Medisch Spectrum Twente, Enschede, the Netherlands	Potters, Jan-Willem Florax, Anna A.
Department of Anesthesiology, Erasmus University Medical Center, Rotterdam, the Netherlands	Harmon, Matthew B.A.
Department of Anesthesiology, Leiden University Medical Center (LUMC), Leiden, the Netherlands	Helmerhorst, Hendrik J.F. Sarton, Elise Y. Laman Trip, Charlotte N.
Department of Anesthesiology, The Netherlands Cancer Institute – Antoni van Leeuwenhoek Hospital, Amsterdam, the Netherlands	Hemmes, Sabrine N.T. Broens, Suzanne
Department of Anesthesiology, Zaans Medical Center, Zaandam, the Netherlands	Koster, Stephanie C.E. Nass, Stefan A.
Department of Anesthesiology, Maasstad hospital, Rotterdam, the Netherlands	Koopman, Joseph S.H.A.
Department of Anesthesiology, Alrijne hospital, Leiderdorp, the Netherlands	van de Wint, Thijs C. Almac, Emre
Department of Anesthesiology, Ospedale San Martino (IRCCS Ospedale Policlinico San Martino), Genoa, Italy	Ball, Lorenzo Pelosi, Paolo Battaglini, Denise Robba, Chiara
Department of Anesthesiology, University Hospital Carl Gustav Carus, Dresden, Germany	Gama de Abreu, Marcelo Wittenstein, Jakob Huhle, Robert
Department of Anesthesiology, Hospital Universitari i Politécnic La Fe, Valencia, Spain	Díaz Cambronero, Oscar Mazzinari, Guido Argente Navarro, Maria P
Department of Anesthesiology, Medizinische Universität Innsbruck, Inssbruck, Austria	Gasteiger, Lukas Staier, Nikolai Mörtl, Maximilian

## Appendix 2

### Definitions of extrapulmonary postoperative complications

#### Sepsis

Defined as life–threatening organ dysfunction caused by a dysregulated host response to infection. Organ dysfunction can clinically be represented by an increase in the Sequential [Sepsis–related] Organ Failure Assessment (SOFA) score of 2 points or more, according to the SEPSIS − 3 definition [37].

#### Septic shock

Identified with clinical construct of sepsis with persisting hypotension requiring vasopressors to maintain MAP ≥ 65 mmHg and having a serum lactate level > 2 mmol/L (18 mg/dL) despite adequate volume resuscitation [37].

#### Extrapulmonary infection

Wound infection and any other infections.

#### Anastomotic leak

The situation of (perceived) failure of an anastomosis leading to leakage of intraluminal substances to the exterior.

#### Acute renal failure

An abrupt (within 48 h) reduction in kidney function, currently defined as an absolute increase in serum creatinine of ≥ 26.4 mcmol/L, a percentage increase in serum creatinine of ≥ 50% (1.5–fold from baseline), or a reduction in urine output (documented oliguria of less than 0.5 ml/kg per hour for more than 6 h. As defined by AKIN [38].

## Appendix 3

**Table 5:** Schedule for enrolment and assessments (SPIRIT 2013 figure)

	**Study period**										
	**Enrollment**	**Allocation**			**Post–allocation**						**Close out**
**Timepoint**	**Preoperative visit**	**Before anesthesia**	**During surgery**	**End of surgery**	**POD 1**	**POD 2**	**POD 3**	**POD 4**	**POD 5**	**Hospital discharge**	**POD 90**
**Enrolment**											
Eligibility screen	X										
Informed consent	X										
Baseline variables	X										
Preoperative vitals	X										
History of previous disease	X										
Lab/chest imaging (facultative)	X										
Allocation		X									
**Interventions**											
Individualized high PEEP with RMs			X								
Low PEEP without RMs			X								
Respiratory/hemodynamic variables			X								
Intraoperative complications			X								
Anesthesia/surgery variables				X							
Need for rescue strategy/protocol deviation or violation				X							
Fluid and transfusion requirements				X							
**Assessments**											
Recovery status/baseline variables/vitals					X	X	X	X	X	X	
Lab/chest imaging (facultative)					X	X	X	X	X	X	
Postoperative pulmonary complications					X	X	X	X	X	X	
Postoperative extrapulmonary complications					X	X	X	X	X	X	
(S)AE					X	X	X	X	X	X	X
Unplanned admission to an ICU					X	X	X	X	X	X	
Length of hospital stay											X
Length of ICU stay											X
QoL questionnaire (only in Dutch patients)											X

## Abbreviations

ACS: Acute Coronary Syndrome
ACLS: Advanced Cardiac Life Support
AKIN: Acute Kidney Injury Network
AMC: Academic Medical Center
ARDS: Acute Respiratory Distress Syndrome
ARISCAT: Assess Respiratory Risk in Surgical Patient in Catalonia
UMC: University Medical Centers
COPD: Chronic Obstructive Pulmonary Disease
DESIGNATION: Driving prESsure durIng GeNeral AnesThesIa fOr open abdomiNal surgery
DSMB: Data Safety Monitoring Board
EDC: Electronic Data Capture
ERAS: Enhanced Recovery After Surgery
FiO_2_: Inspired oxygen fraction
GENERATOR: DrivinG prEssure duriNg gEneRal AnesThesia fOr minimally invasive abdominal suRgery
ICU: Intensive Care Unit
I:E: Inspiratory to expiratory ratio
MAP: Mean Arterial Pressure
METC: Medical Ethical Committee
NYHA: New York Heart Association
PBW: Predicted Body Weight
PEEP: Positive End–Expiratory Pressure
P_plat_: Plateau Pressure
PPCs: Postoperative Pulmonary Complications
RCT: Randomized Clinical Trial
RM: Recruitment Maneuver
TIVA: Total IntraVenous Anesthesia
TOF: Train-Of-Four
VAS: Visual Analog Scale
V_T_: Tidal Volume
ZonMw: The Netherlands Organization for health Research and Development
ΔP: Driving Pressure
